# Development of Human Serum Albumin-Based Hydrogels for Potential Use as Wound Dressings

**DOI:** 10.3390/gels12010064

**Published:** 2026-01-09

**Authors:** Inna Zharkova, Irina Bauer, Oksana Gulyaeva, Evgenia Kozyreva, Zhanna Nazarkina, Elena Dmitrienko

**Affiliations:** Institute of Chemical Biology and Fundamental Medicine of the Siberian Branch of the Russian Academy of Sciences, 630090 Novosibirsk, Russia; malbakhova.inna@yandex.ru (I.Z.); bauer@1bio.ru (I.B.); gulyaeva00ks@gmail.com (O.G.); malova.ev.an@gmail.com (E.K.); zha_naz@1bio.ru (Z.N.)

**Keywords:** wound management, human serum albumin, HSA-based hydrogels, protein-based materials, hydrogel formation

## Abstract

Protein-based materials such as human serum albumin (HSA) have demonstrated significant potential for the development of novel wound management materials. For the first time, the formation of HSA-based hydrogels was proposed using a combination of thermal- and ethanol-induced approaches. The combination of phosphate-buffered saline (PBS) and limited (up to 20% *v*/*v*) ethanol content offers a promising strategy for fabricating human serum albumin-based hydrogels with tunable properties. The hydrogel formation was studied using in situ dynamic light scattering (DLS) for qualitative and semi-quantitative analysis of the patterns of protein hydrogel formation through thermally induced gelation. The rheological properties of human serum albumin-based hydrogels were investigated. Hydrogels synthesized via thermally induced gelation using a denaturing agent exhibit a dynamic viscosity ranging from 100 to 10,000 mPa·s. The biocompatibility, biodegradability, and structural stability of human serum albumin-based hydrogels were comprehensively evaluated in physiologically relevant media. These human serum albumin-based hydrogels represent a promising platform for developing topical therapeutic agents for wound management and tissue engineering applications. This study investigated the kinetics of tetracycline release from human serum albumin-based hydrogels in PBS and fetal bovine serum (FBS). All tested formulations of HSA-based hydrogels loaded with tetracycline (1 mg/mL) demonstrated antibacterial activity against *Staphylococcus aureus*, *Staphylococcus epidermidis*, *Staphylococcus haemolyticus*, and *Corynebacterium striatum* strains.

## 1. Introduction

The skin, the biggest organ in the human body, acts as a critical barrier that maintains homeostasis and protects internal systems from adverse environmental factors [1], while constant exposure to external threats renders it susceptible to various wound injuries [2]. Moreover, wound healing is a multifaceted process involving multiple stages, each requiring stage-specific therapeutic strategies, particularly in the case of chronic wounds [3]. Conventional wound dressings, such as gauze, cotton wool, and bandages, while providing protection against external contaminants, fail to establish optimal moist wound healing conditions, which is crucial for the initial stages of tissue regeneration [4]. Moreover, their drying and subsequent removal cause mechanical re-injury to the wound bed, thereby inducing patient discomfort and significantly impeding the re-epithelialization process [5]. Furthermore, conventional dressings lack targeted therapeutic effects and exhibit inherent functional limitations, substantially restricting their efficacy in the management of complex wounds.

Of particular promise for wound management are protein-based hydrogels and their derivatives [6], which offer significant advantages, including flexibility, high hygroscopicity, and the ability to adapt to varying environmental conditions (e.g., temperature, pH, enzyme presence). These properties contribute to their efficacy across all stages of the wound healing process [7]. A critical property of protein-based hydrogels is their tunable functionality—the ability to dynamically modulate their properties in response to wound microenvironment changes, thereby accelerating or decelerating specific healing phases. Furthermore, they serve as scaffolds that facilitate cellular migration, proliferation, and tissue regeneration [8]. The development of biocompatible and biodegradable hydrogels from natural proteins, such as fibrillar (e.g., collagen, elastin, silk fibroin) and globular (e.g., albumin) types, represents a promising research direction due to their wide availability and well-characterized properties [9]. Hydrogels derived from collagen, elastin, and silk fibroin, which are widely used in wound management and regenerative medicine, exhibit high affinity for epithelial tissues, hydrophilicity, biocompatibility, and biodegradability. However, their widespread integration is hindered by suboptimal mechanical properties, as well as the complexity and high cost of purification processes [10]. Globular proteins are capable of forming hydrogels under specific pH or temperature conditions or in the presence of cross-linking agents. These hydrogels exhibit exceptional mechanical strength, stability, and tunability, making them promising materials for applications in biotechnology and medicine [11].

One of the most prominent examples of globular proteins in biomedicine are bovine serum albumin (BSA) and human serum albumin (HSA), which demonstrate exceptional biological functionalities. These include promoting angiogenesis, enhancing capillary permeability, facilitating wound healing, and supporting bone regeneration [10]. Moreover, serum albumin is a naturally occurring transport protein that possesses multiple ligand binding sites [12]. The role of the carrier is to increase the circulation time of ligands in the body, reduce renal elimination, increase the solubility of hydrophobic drugs and reduce its toxicity [13]. In view of the bioresorbable properties of albumin-based hydrogels [9], it can be anticipated that, upon loading a therapeutic agent into the gel, it will be released in complex with the protein. Consequently, the concomitant release of albumin with the drug loaded into the gel from the resorbable hydrogel can provide a combination of controlled local and systemic release and improved pharmacokinetic properties of drugs. In recent years, there has been growing interest in the use of BSA- and HSA-based hydrogels for the treatment of wounds of various etiologies. However, current research in this area predominantly involves composite systems combining these proteins with carbohydrates or synthetic polymers [11,14,15,16,17]. These hydrogels need labor-intensive fabrication processes, necessitating the development of alternative approaches enabling the production of albumin-based hydrogels with enhanced simplicity and versatility.

There are four principal methodologies for fabricating albumin-based hydrogels: pH-induced gelation, thermally induced assembly, chemically cross-linking agents, and the application of hydrophobic interaction-modifying agents for protein macromolecules [18,19,20]. Thermally induced gelation represents a versatile, rapid, and convenient method for fabricating albumin-based hydrogels while eliminating the need for toxic and/or costly cross-linking agents [21]. Nevertheless, the majority of studies on thermally induced albumin hydrogel formation have been conducted using BSA, despite the fact that BSA and human serum albumin (HSA) share only 76% amino acid sequence homology [22]. This substantial divergence in primary structure results in significant functional differences between the two proteins. Consequently, data obtained from BSA-based systems cannot be directly extrapolated to HSA behavior or performance. It is important to note that during thermally induced gelation, both BSA and HSA form gels within the temperature range of 70 °C to 100 °C [23,24,25], which exceeds their denaturation thresholds. This, in turn, leads to several critical issues: impairment of albumin’s binding sites for therapeutic agents, hydrogel opacity, and reduced cell proliferation on the hydrogel surface, among others [22]. In addition to temperature and protein concentration, various chemical agents significantly influence the albumin gelation process. For instance, urea [26] and surfactants [27] substantially reduce the gelation temperature and time for BSA by promoting protein unfolding and disrupting hydrophobic interactions. Furthermore, several studies have reported the formation of BSA-based hydrogels induced by ethanol (EtOH) at a temperature of 37 °C [28,29]. Moreover, it has been demonstrated that small quantities of ethanol can reduce the gelation temperature of BSA below 59 °C [29]. However, to the best of the authors’ knowledge, no studies have been conducted to date on the influence of ethyl alcohol on the thermally induced formation of HSA-based hydrogels. Thus, the present study will fabricate human serum albumin-based hydrogels via thermal gelation with ethanol addition, which represent promising materials for developing advanced hydrogel wound dressings.

## 2. Results and Discussion

### 2.1. Effect of Synthesis Conditions on Human Serum Albumin-Based Hydrogel Formation

Despite extensive research on serum albumin (both bovine and human) as a drug-delivery vehicle [30,31,32,33], its application in hydrogel-based biomedical systems has only recently emerged. Consequently, the mechanisms governing albumin hydrogel formation remain inadequately characterized. Nevertheless, interest in these materials is growing due to their biocompatibility, bioinertness, and capacity for gelation across a broad concentration range, making them suitable for tissue engineering and regenerative medicine [34,35]. Thermally induced gelation is a versatile and practical method for fabricating albumin-based hydrogels [22]. In terms of structure, this process occurs via two sequential stages: first, thermal denaturation unfolds albumin’s polypeptide chains, disrupting its tertiary structure [36]; second, progressive heating reduces α-helical content while promoting intra- and intermolecular β-sheet formation, which constitutes the three-dimensional hydrogel scaffold [37]. Thus, thermal gelation enables rapid, straightforward fabrication of stable hydrogels without toxic chemical cross-linkers [21].

**Figure 1:**
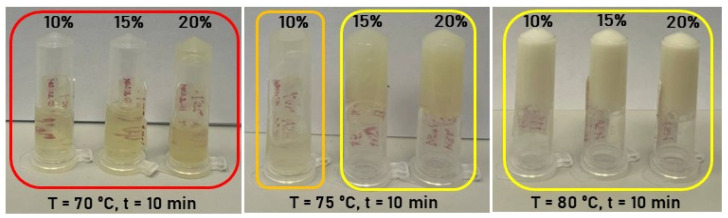
Photographs of HSA hydrogels in aqueous solution as a function of protein concentration. Red border denotes conditions yielding no gelation; orange border indicates incomplete gelation; yellow border signifies hydrogel formation with substantial HSA denaturation.

**Figure 2:**
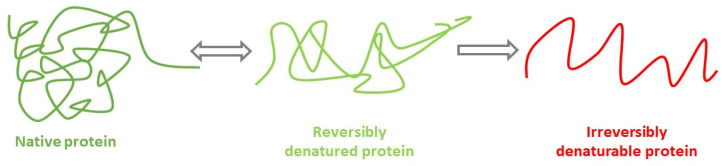
Schematic representation of protein hydrogel formation via thermally induced gelation according to the Eyring—Lumry model.

In the initial series of experiments, human serum albumin hydrogels were fabricated via thermally induced gelation of aqueous HSA solutions. Hydrogel samples with HSA concentrations of 10–20% (*w*/*v*) were synthesized in an aqueous medium at 60–80 °C with a 10 min incubation period. Data presented in Table 1 and Figure 1 demonstrate that hydrogels containing 20% (*w*/*v*) HSA exhibit incomplete gelation at 70 °C. At temperatures ≥ 75 °C, turbidity develops due to albumin denaturation. According to established literature, thermal protein denaturation follows the Eyring—Lumry model [38,39] (Figure 2), wherein the first stage entails reversible conformational changes [40], while the second stage involves irreversible structural alterations. For HSA, such irreversible changes occur predominantly at temperatures exceeding 74 °C [40,41]. Consequently, gelation above 74 °C leads to substantial loss of optical transparency in the resultant hydrogels, attributable to extensive irreversible denaturation of albumin molecules. Optical transparency is critical for wound-healing hydrogels, as it permits visual monitoring of wound progression without frequent dressing removal—a procedure that causes significant patient distress. Additionally, albumin denaturation compromises drug-binding sites, thereby limiting the utility of albumin-based scaffolds in advanced drug delivery applications [42].

In the preparation of albumin-based hydrogels, ethanol is commonly utilized to induce the denaturation and subsequent gelation of HSA [9]. Although the precise mechanisms by which ethanol modulates serum albumin structure remain debated, prevailing theory posits that this less polar solvent disrupts hydrophobic interactions within the protein, promoting interchain hydrogen bonding and a conformational shift toward β-sheet-rich extended structures [43]. Prior investigations of ethanol-mediated gelation have focused exclusively on BSA in aqueous-ethanol systems, demonstrating that gelation occurs below BSA’s thermal denaturation temperature following ethanol addition [28]. Notably, the influence of ethanol on the structural and gelation properties of HSA-hydrogels remains unexplored in the literature.

**Figure 3:**
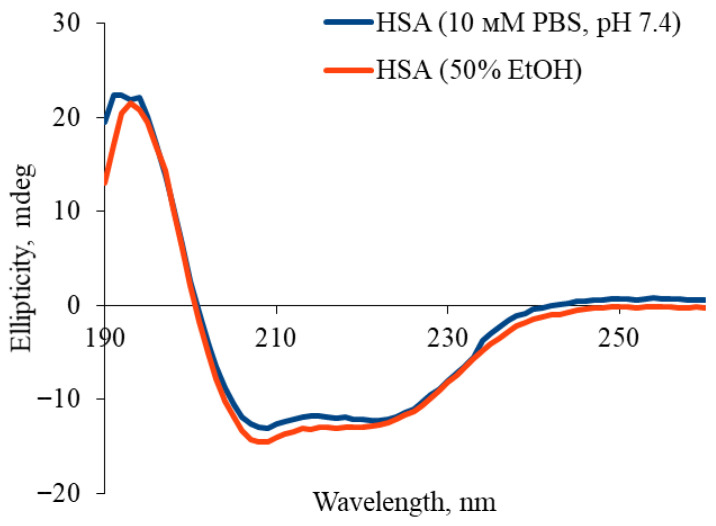
Circular dichroism spectra for HSA in 10 mM PBS and in 10 mM PBS/50% EtOH.

Analysis of circular dichroism (CD) spectra revealed that ethanol addition altered the secondary structure of human serum albumin (HSA), characterized by reduced amplitude of negative bands at 208 nm and 222 nm which indicates a partial loss of α-helical structure (Figure 3). Quantitative deconvolution (Table 2) confirmed a decrease in α-helical content from 61.1% ± 0.9% to 54.3% ± 0.8%, accompanied by a concomitant increase in β-sheet content from 3.2% ± 0.1% to 5.6% ± 0.2%. These changes reflect partial, reversible reorganization of HSA’s secondary structure in ethanol-water mixtures, while retaining key native spectral features. Critically, under these conditions (≤50% ethanol), the protein maintains partial native conformation, consistent with literature demonstrating that globular proteins retain ~50% α-helical structure at 40–50% ethanol. Irreversible denaturation and aggregation dominate only at higher ethanol concentrations (≥60–70%) [44]. Thus, 50% ethanol represents an optimal compromise for HSA hydrogel fabrication, enabling sufficient structural modification for gelation while preserving the native conformation essential for functional applications.

**Figure 4:**
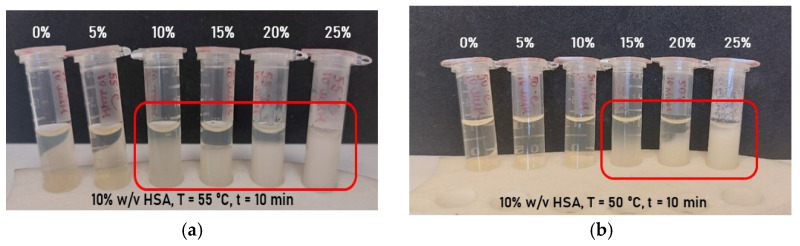
Photographs of aqueous-based hydrogels as a function of ethanol volume fraction: (**a**) hydrogels formed at 55 °C; (**b**) hydrogels formed at 50 °C. Phase separation is highlighted with red frames, and volume fractions of ethanol are labeled in white font.

Hydrogel samples containing 10% (*w*/*v*) human serum albumin (HSA) in aqueous medium were synthesized at 50–55 °C with a 10 min incubation period, incorporating varying ethanol volume fractions (Figure 4a,b). Ethanol addition significantly reduced the HSA gelation temperature from 80 °C to 55 °C for 10% (*w*/*v*) protein, but resulted in hydrogels with poor mechanical stability. Over time, these samples exhibited phase separation and precipitation of denatured HSA. Consequently, these findings demonstrate that buffered aqueous solutions of defined ionic strength and pH are necessary for stable HSA-hydrogels with preserved native protein structure.

**Figure 5:**
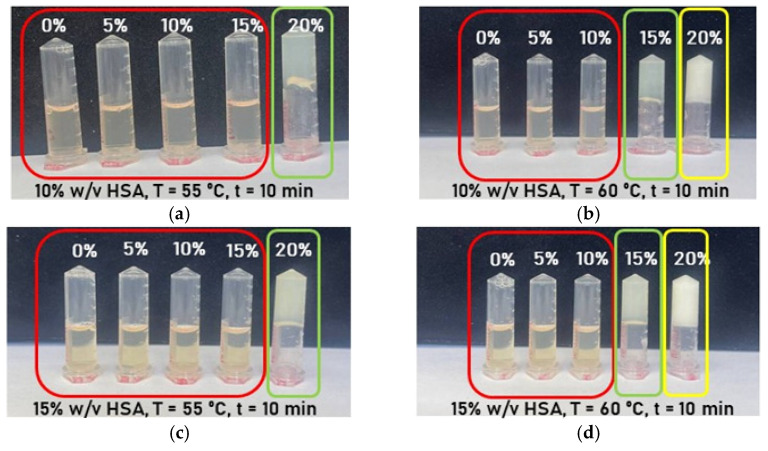

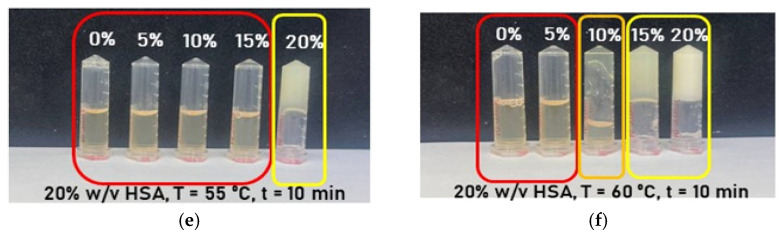
Photographs of aqueous-based hydrogels as a function of ethanol volume fraction: (**a**) hydrogels formed at 55 °C with 10% *w*/*v* HSA; (**b**) hydrogels formed at 55 °C with 15% *w*/*v* HSA; (**c**) hydrogels formed at 55 °C with 20% *w*/*v* HSA; (**d**) hydrogels formed at 50 °C with 10% *w*/*v* HSA; (**e**) hydrogels formed at 50 °C with 15% *w*/*v* HSA; (**f**) hydrogels formed at 50 °C with 20% *w*/*v* HSA. The red frame corresponds to unformed hydrogel; the orange frame indicates partially formed hydrogel; the yellow frame represents hydrogel formed with substantial denaturation of HSA; green frames indicate formed hydrogel.

Subsequent experiments on the formation of human serum albumin-based hydrogels were conducted using phosphate-buffered saline and ethanol with a volume fraction not exceeding 20% (*v*/*v*) in the mixture. The choice of buffer was motivated by the fact that phosphate-buffered saline is approved for medical applications, while the ethanol volume fraction was limited based on the following considerations. The study [45] demonstrated that the process of conformational changes in the HSA structure under the influence of ethanol is non-linear: free water molecules or weakly hydrogen-bonded water molecules predominate at low ethanol concentrations (20% (*v*/*v*)), whereas the hydration layer transforms into a strongly hydrogen-bonded water cluster as the ethanol concentration increases. These water clusters play a substantial role in linking individual protein molecules, which may be necessary for nucleation and protein precipitation, while excessive ethanol addition can disrupt the HSA structure. Therefore, to preserve the protein structure as close as possible to its native state, the volume fraction of ethanol in the hydrogel was maintained at or below 20% (*v*/*v*). The data presented in Figure 5 and Table 3 demonstrate that the combination of PBS and ethanol allowed hydrogels with both optical transparency and mechanical stability to develop. The authors identified the following compositions as optimal: 10% HSA–20% EtOH and 15% HSA–20% EtOH for gelation at 55 °C with a 10 min incubation; 10% HSA–15% EtOH and 15% HSA–15% EtOH for gelation at 60 °C with a 10 min incubation.

The influence of incubation time on the formation of HSA-based hydrogels was investigated. For HSA-PBS-ethanol systems that did not form a hydrogel within 10 min, the incubation time was extended to 30 and 60 min at their respective gelation temperatures. For samples that formed a hydrogel with substantial denaturation within 10 min, the incubation time was reduced to 1 and 5 min. It was demonstrated that extending the heating time for hydrogels previously unformed within 10 min resulted in successful gelation only at 20% ethanol volume fraction in the mixture (Table 4). Conversely, reducing the heating time for hydrogels exhibiting substantial denaturation after a 10 min incubation proved to be an effective approach, except for mixtures with a 20% ethanol volume fraction (Table 5).

**Figure 6:**
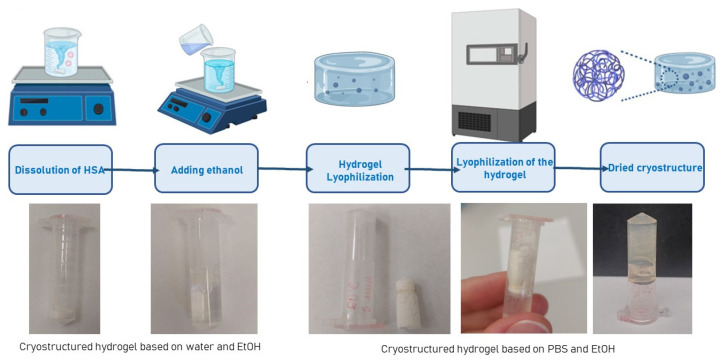
Lyophilization scheme of human serum albumin-based hydrogels and photographs of the resulting cryotextured structures before and after resuspension.

Screening experiments on the lyophilization of human serum albumin-based hydrogels were conducted. Freeze-drying is a versatile technique for producing porous hydrogel matrices, which have recently attracted growing interest in tissue engineering. Moreover, the lyophilization of hydrogels can significantly extend their shelf life, particularly when they are loaded with various therapeutic agents. It was demonstrated that during the lyophilization of human serum albumin-based hydrogels with water, the freeze-drying process produced a powder (Figure 6). This powder could be resuspended into a solution upon the addition of water. In contrast, hydrogels based on HSA and PBS were lyophilized into a single, pellet-like structure. When water was added to this structure, it re-suspended and re-formed a hydrogel. Thus, the developed approach for fabricating human serum albumin-based hydrogels opens up broad prospects for the further investigation of porous hydrogel scaffolds derived from them.

**Figure 7:**
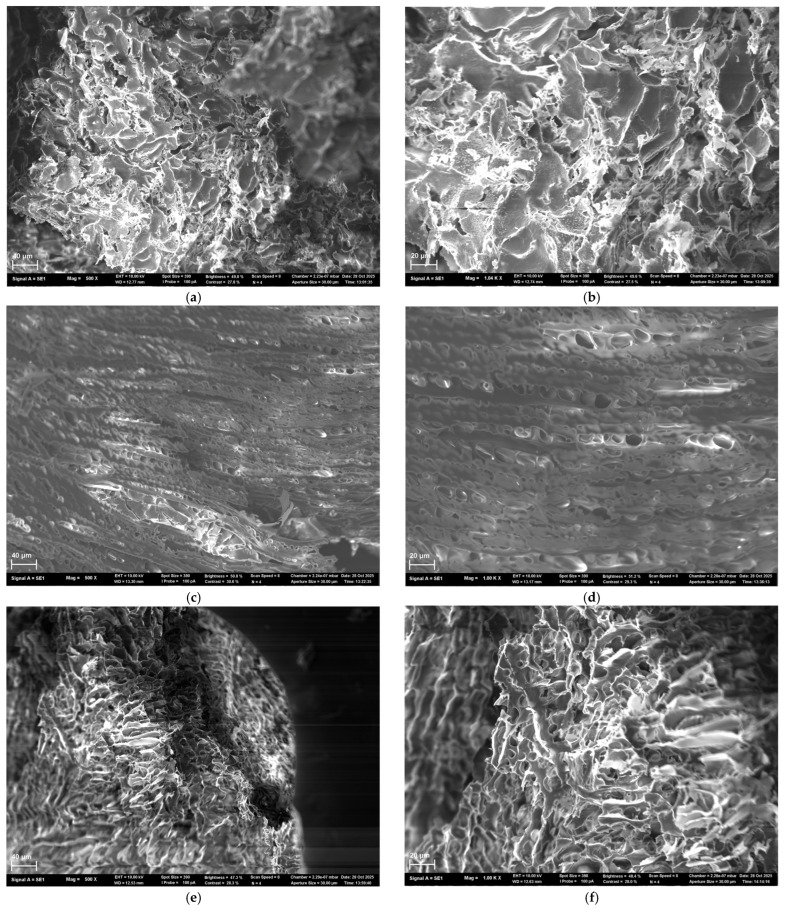
SEM micrographs of lyophilized human serum albumin-based scaffolds: (**a**) 15 HSA–15 EtOH, ×500; (**b**) 15 HSA–15 EtOH, ×1000; (**c**) 20 HSA–15 EtOH, ×500; (**d**) 20 HSA–15 EtOH, ×1000; (**e**) 15 HSA–10 EtOH, ×500; (**f**) 15 HSA–10 EtOH, ×1000.

Scanning electron microscopy (SEM) was employed to analyze the morphology of lyophilized scaffolds prepared from hydrogels of three compositions: 15 HSA–15 EtOH, 20 HSA–15 EtOH, and 15 HSA–10 EtOH. For the 15 HSA–15 EtOH composition (Figure 7a,b), a porous structure with irregularly shaped pores ranging from several to tens of micrometers in size is observed. Well-defined boundaries between material fragments indicate spatial heterogeneity in polymer network density, reflecting regions with varying degrees of packing. At higher magnification, thin, interwoven fibrillar elements are revealed, forming an open, highly interconnected porous network. Some areas exhibit increased density, while others display pronounced inter-fibrillar porosity, confirming the multiscale, sponge-like nature of the scaffold architecture. Scaffolds of the 20 HSA–15 EtOH composition (Figure 7c,d) exhibit a distinct layered, oriented microstructure. Numerous oval- and slit-shaped pores (1–10 µm in size) are present between the layers. Despite a degree of ordering, the structure remains irregular, suggesting spontaneous self-organization of the protein network during gelation. The 15 HSA–10 EtOH scaffold (Figure 7e,f) displays a hierarchical porous morphology: at the macro-scale, fragmented domains with a “foamy” texture and visible cracks indicate dehydration-induced shrinkage and brittleness in the dry state. At higher magnification, an open, percolating pore network emerges, composed of irregularly shaped pores (from rounded to slit-like), bounded by thin walls (1–5 µm thick) with granular surface texture—indicative of secondary microstructure and composite nature, combining dense, scaffold-like regions with more loosely packed, spongy zones. Collectively, SEM analysis reveals a clear compositional dependence of scaffold morphology: decreasing the HSA mass fraction and increasing the ethanol volume fraction in the precursor solution lead to enhanced porosity and the development of a more open, less densely packed fibrous architecture.

### 2.2. Investigation of Hydrogel Gelation Kinetics via Dynamic Light Scattering

To determine the influence of ethanol and the aqueous environment on the gelation kinetics of HSA-based hydrogels, in situ hydrogel formation tests were conducted with simultaneous monitoring by DLS. Although the microstructure and mechanical properties of protein- and peptide-based hydrogels have been extensively characterized using techniques such as rheometry and transmission electron microscopy, the kinetics of aggregation and subsequent gelation have received significantly less attention [46]. Hydrogel formation proceeds through the following stages: (1) Initial homogeneous solution; (2) Aggregation of colloidal particles into clusters; (3) Cluster growth; (4) Percolation and hydrogel network formation; (5) Development of a macrogel network [47]. DLS enables real-time observation of all these stages during hydrogel formation, allowing determination of the percolation time and average cluster size.

**Figure 8:**
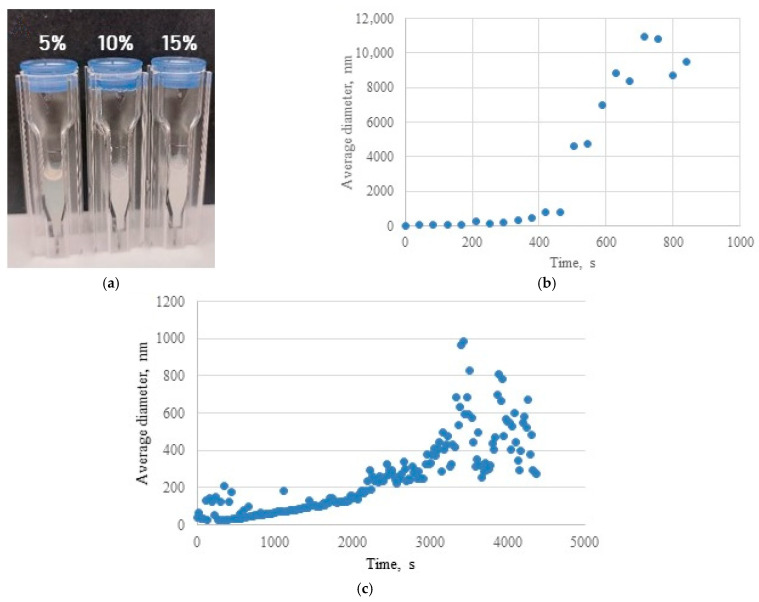
(**a**) Hydrogels in a DLS cuvette in aqueous solutions with varying ethanol volume fractions (values indicated in white); (**b**) gelation kinetics monitored by DLS for 10% (*w*/*v*) HSA in 10% (*v*/*v*) ethanol/water at 60 °C; (**c**) gelation kinetics monitored by DLS for 10% (*w*/*v*) HSA in 15% (*v*/*v*) ethanol/PBS at 60 °C.

In situ formation of HSA-based hydrogels was performed in a DLS cuvette with simultaneous monitoring. The visual appearance of hydrogels formed in the cuvette, as a function of ethanol volume fraction, is shown in Figure 8a. When HSA was dissolved in water, hydrogel formation occurred exclusively in the absence of ethanol; even 5% (*v*/*v*) ethanol induced gelation. Post-gelation, the average hydrodynamic diameter exceeded 1000 nm (Table 6). In contrast, when HSA was diluted in phosphate-buffered saline, hydrogel formation required a higher ethanol volume fraction (≥15%). Under these conditions, the average hydrodynamic diameter after gelation was substantially smaller (400–500 nm). These results demonstrate that stabilization of ionic strength and pH is essential for forming sedimentation-resistant hydrogels during thermally induced gelation. PBS addition reduced the average aggregate size of HSA, yielding stable, optically transparent hydrogels.

It is important to note the limitations of DLS for the hydrogel systems investigated here. The upper size detection limit is constrained by sedimentation onset [48]. For aqueous-based hydrogels, kinetic curves exhibited classical sigmoidal profiles (Figure 8). In PBS-based systems, significant fluctuations in hydrodynamic radius occurred post-gelation (Figure 8c) due to contributions from depolarized light scattering by clusters/percolation networks and multiple light scattering effects [49]. Consequently, aqueous-based hydrogels with limited three-dimensional network robustness and high susceptibility to sedimentation were detected primarily as microparticles. For strongly cross-linked PBS-based hydrogels, multiple scattering introduced substantial errors in mean cluster size and cross-linking density determinations. Nevertheless, despite significant error margins in absolute values during kinetic profiling, this in situ method enables qualitative and semi-quantitative assessment of thermally induced protein hydrogel formation mechanisms.

### 2.3. Investigation of Rheological Properties of HSA-Based Hydrogels

The rheological properties of HSA-based hydrogels were systematically investigated to evaluate their suitability for biomedical applications, including injectable drug delivery and tissue engineering. Dynamic viscosity was assessed at shear rates of 3, 15, and 45 s^−1^ (Table 7). Most hydrogels exhibit non-Newtonian pseudoplastic behavior (shear-thinning), which is characteristic of thixotropic systems [50]. This property is critical for applications requiring injectability, controlled drug delivery, or bioprinting in tissue engineering. The rheological properties of human serum albumin-based hydrogels, namely flow behavior, dynamic viscosity, and thixotropy, were investigated in relation to the hydrogel synthesis conditions.

Based on dynamic viscosity measurements, the hydrogels were grouped into four distinct categories (Table 8):

- Group 1 (100–1000 mPa·s at 3 s^−1^) includes formulations with lower HSA and ethanol concentrations (e.g., 10% HSA with 10–15% EtOH, incubated 30–60 min). These systems demonstrate low resistance to flow and are perfect candidates for minimally invasive delivery, aligning with viscosity thresholds reported for injectable hydrogels in the literature [51,52].
- Group 2 (1000–5000 mPa·s at 3 s^−1^) encompasses moderately crosslinked systems, such as 15% HSA with 10–15% EtOH or 20% HSA with 10–15% EtOH (short incubation times). These hydrogels retain injectability while offering enhanced mechanical stability post-injection, making them suitable for sustained release applications.
- Group 3 (5000–10,000 mPa·s at 3 s^−1^) includes formulations with higher protein content and intermediate ethanol concentrations (e.g., 15–20% HSA with 15–20% EtOH, 5–10 min incubation). Their elevated viscosity suggests potential use as structural scaffolds or depot-forming carriers for prolonged therapeutic action, though injection may require larger-bore needles or pre-warming to reduce viscosity.
- Group 4 (10,000–25,000 mPa·s at 3 s^−1^) comprises systems with high ethanol content (20% EtOH) and moderate HSA concentrations (10–15%). Notably, while some samples in this group exhibited extremely high viscosities (e.g., 10 HSA–20 EtOH at 10 min: ~32,070 mPa·s at 5 s^−1^), others (e.g., 15 HSA–20 EtOH at 10 min) became unmeasurable, likely due to excessive protein denaturation and rapid gelation, leading to solid-like behavior that exceeds the rheometer’s detection range. This highlights the narrow formulation window for ethanol-induced HSA gelation: while ethanol promotes crosslinking via partial denaturation, excessive concentrations or prolonged exposure disrupt network homogeneity, resulting in brittle or non-processable gels.

The thermally induced gelation method supplemented with ethanol as an additional denaturing agent offers a key advantage: it enables the production of stable hydrogels across a wide viscosity range (100–10,000 mPa·s). This versatility allows for the development of universal platforms—from relatively fluid formulations suitable for topical application on superficial wounds to highly viscous hydrogels for forming therapeutic coatings in deep tissue injuries. In conclusion, the proposed approach to forming HSA hydrogels combines simplicity, high reproducibility, and flexibility in tuning physico-mechanical properties, rendering it a promising strategy for applications in tissue engineering and regenerative medicine.

**Figure 9:**
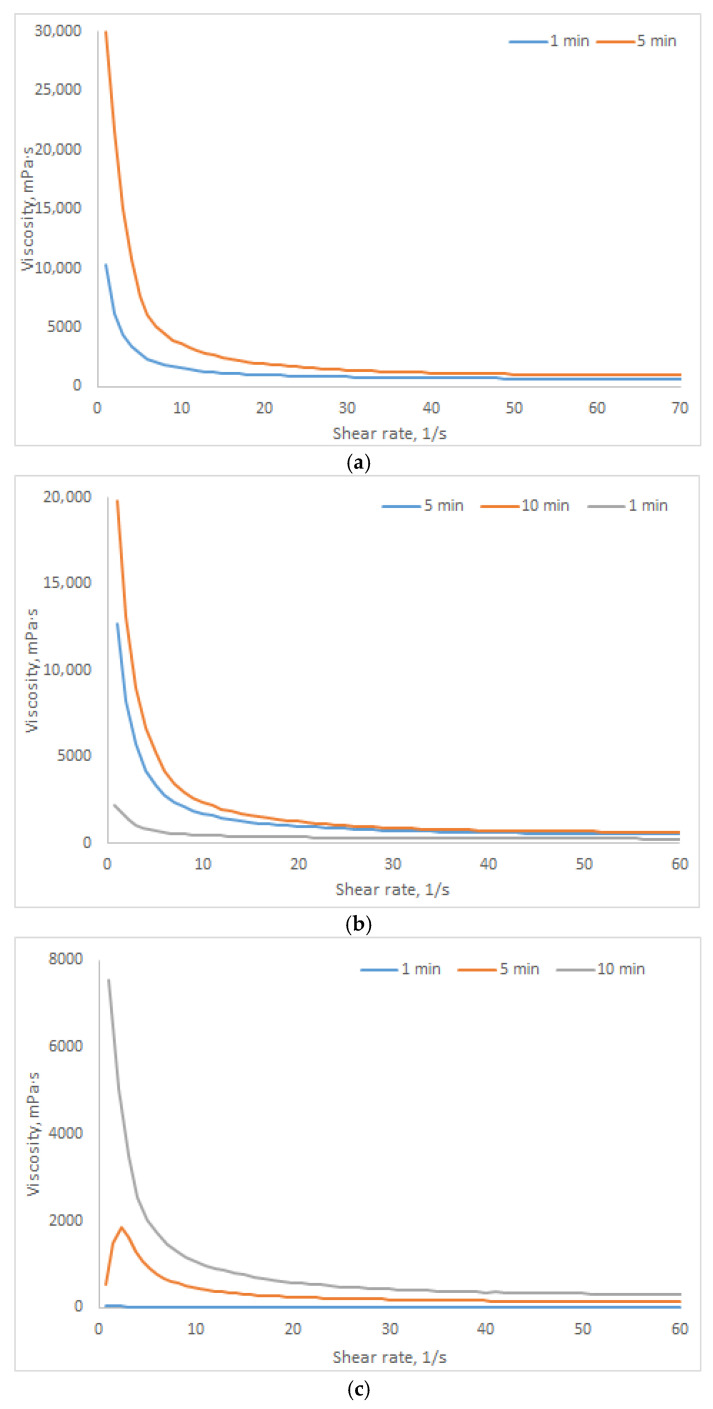
Dynamic viscosity curves for hydrogels with 15% *v*/*v* ethanol: 10% *w*/*v* HSA (**a**) 15% *w*/*v* HSA (**b**); 20% *w*/*v* HSA 10% (**c**).

Figure 9 presents the rheological curves of dynamic viscosity for HSA-based hydrogels with concentrations of 10–20% (*w*/*v*) across a shear rate range of 1–60 s^−1^, at a fixed ethanol volume fraction of 15% (*v*/*v*). All formulations exhibit pronounced pseudoplastic behavior, a characteristic feature of physically crosslinked protein-based systems, manifested as a decrease in viscosity with increasing shear rate due to the transient disruption of the gel network under mechanical stress. With increasing incubation time, a consistent increase in viscosity is observed across the entire shear rate range, indicating progressive reinforcement of the gel matrix due to the development of intermolecular interactions and the formation of a denser, more ordered three-dimensional network. This process is kinetically controlled: incubation time emerges as a critical parameter governing the extent of crosslinking and the final rheological properties of the hydrogel. Moreover, the rate and extent of gelation are directly correlated with HSA concentration: systems with higher HSA content (20% *w*/*v*) reach maximum viscosity more rapidly and exhibit more pronounced structural evolution compared to more dilute formulations (10% *w*/*v*), consistent with the data presented in Table 7. Combined analysis reveals that ethanol concentration and incubation time act synergistically to modulate the viscoelastic architecture of the hydrogels. Higher ethanol content accelerates protein denaturation and promotes the formation of intermolecular β-sheet structures, thereby triggering early percolation and rapid development of a rigid network. Conversely, reducing ethanol concentration or extending incubation time enables smoother, more controllable gelation kinetics, allowing for targeted modulation of the material’s mechanical properties for specific biomedical applications.

As shown in Table 9, an increase in the ethanol volume fraction in human serum albumin (HSA)-based hydrogels correlates with a rise in their thixotropic index. This behavior is attributed to the formation of additional hydrophobic associations within the polymer network, driven by the tendency of nonpolar groups to minimize contact with the aqueous environment. These associations act as transient crosslinking points, contributing to mechanical energy dissipation [53]. Unlike covalent bonds, hydrophobic interactions are characterized by lability, reversibility, and cooperativity. Under shear stress, these associations break, leading to gel thinning (transition to a sol-like state). Upon removal of stress, thermodynamically favored reassociation of hydrophobic domains enables self-recovery of the network structure. An increased density of hydrophobic associations accelerates the kinetics of reorganization and enhances the strength of the equilibrium network, resulting in a more pronounced thixotropic response.

Conversely, the decrease in the thixotropic index with increasing HSA concentration (Table 9) reflects a structural transition in the hydrogel network: from a dynamic, physically crosslinked system to a more static matrix exhibiting covalent-like characteristics. In diluted systems, gelation is governed by reversible physical interactions—hydrophobic associations, hydrogen bonding, and electrostatic interactions. These interactions possess short relaxation times, allowing the network to break under shear and rapidly recover upon stress removal, which manifests as high thixotropy. The low crosslinking density ensures sufficient chain mobility for efficient restructuring. In concentrated systems, intense intermolecular aggregation leads to the formation of a dense network stabilized by cooperative interactions, primarily associated with fibrillar aggregates. The elevated crosslinking density and steric constraints drastically reduce macromolecular mobility, slowing down the kinetics of bond rupture and reformation even when the interactions remain physical in nature. Disruption of critical crosslinking points in such networks requires substantial energy due to the high dissociation barrier of cooperative aggregates. Consequently, higher shear stress is needed to induce gel thinning, and structural recovery after stress removal may be incomplete or significantly delayed.

### 2.4. Investigation of the Biocompatibility and Biodegradability of HSA-Based Hydrogels

**Figure 10:**
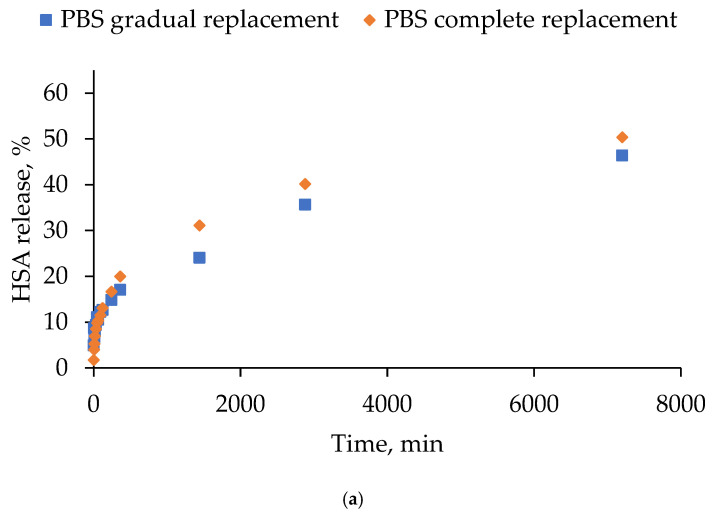

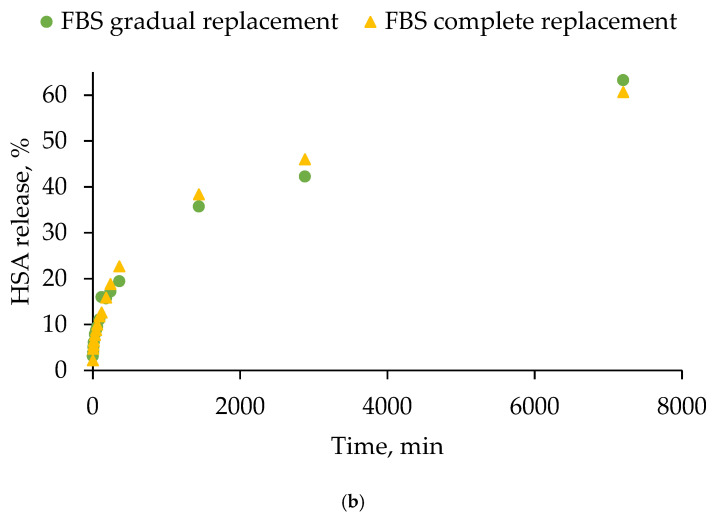
Release curves of HSA from human serum albumin-based hydrogels: (**a**) in PBS; (**b**) in FBS.

The stability of the human serum albumin-based hydrogels was studied in two media (PBS and 10% FBS) under different modes of medium replacement over the hydrogel surface (gradual and complete). Figure 10 presents the HSA release curves in two different media: PBS (Figure 10a) and FBS (Figure 10b). The data points on the graphs represent the total percentage of the HSA released from the hydrogels at the corresponding time point. The HSA release profile from the hydrogels exhibited a biphasic profile under all of the tested conditions. This profile was characterized by an initial rapid release during the first hour, followed by a more gradual sustained release. Across both replacement modes, the final percentage of the HSA release was found to be slightly higher in 10% FBS compared to PBS, suggesting that serum components may partially destabilize the hydrogel network or enhance albumin mobility. The replacement of the medium had a negligible effect on the overall release kinetics, with both approaches yielding highly similar profiles and converging towards comparable plateau levels at later time points.

**Figure 11:**
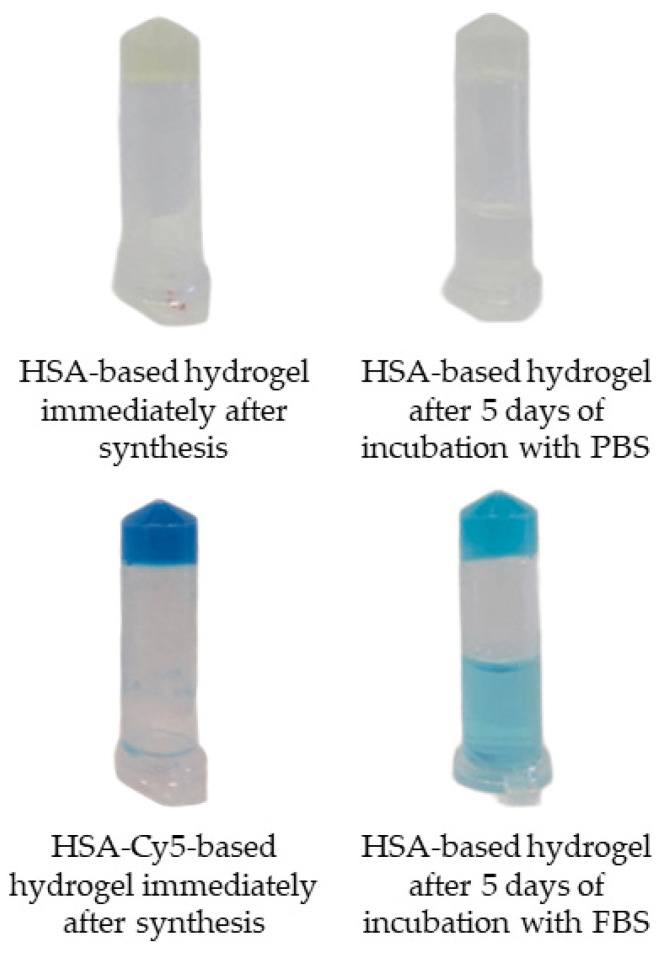
Stability and degradation behavior of HSA-based hydrogels.

Subsequent to a five-day incubation period at 37 °C, with gentle stirring at a rate of 300 rpm, human serum albumin-based hydrogels demonstrated no significant alterations in appearance, depending on the medium used (Figure 11). In both media, the hydrogels largely retained their structural integrity, remaining relatively transparent and compact. This indicates good structural stability and resistance to swelling or disintegration, highlighting their potential for applications requiring stable protein-based scaffolds.

**Figure 12:**
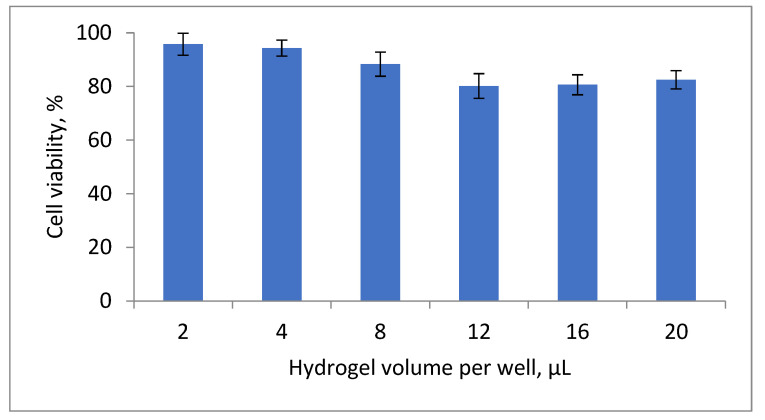
Graphical representation of the MTT test of fibroblast cells after incubation with different concentrations of HSA-hydrogel.

The MTT assay was performed to evaluate the biocompatibility of HSA-based hydrogels with fibroblasts (MRC-5 cell line) at various hydrogel volumes per well (2–20 µL) (Figure 12). As shown in Figure 12, all tested volumes supported high cell viability, indicating no significant cytotoxicity under the experimental conditions. At hydrogel volumes up to 8 µL, cell viability was 94.2 ± 1.8%, 92.5 ± 2.1%, and 87.6 ± 3.3%, respectively. A slight decrease in viability was observed at higher volumes (12–20 µL), ranging from 79.4 ± 4.1% to 82.1 ± 3.7%. This moderate reduction is likely attributable to the higher residual ethanol content, which may exert cytotoxic effects, or to physical constraints associated with testing larger hydrogel volumes in a single well. Importantly, no statistically significant loss of cell viability was observed across the entire range of tested volumes, confirming the biocompatibility of HSA-based hydrogels and supporting their potential as safe platforms for wound healing applications without compromising cellular functions.

**Figure 13:**
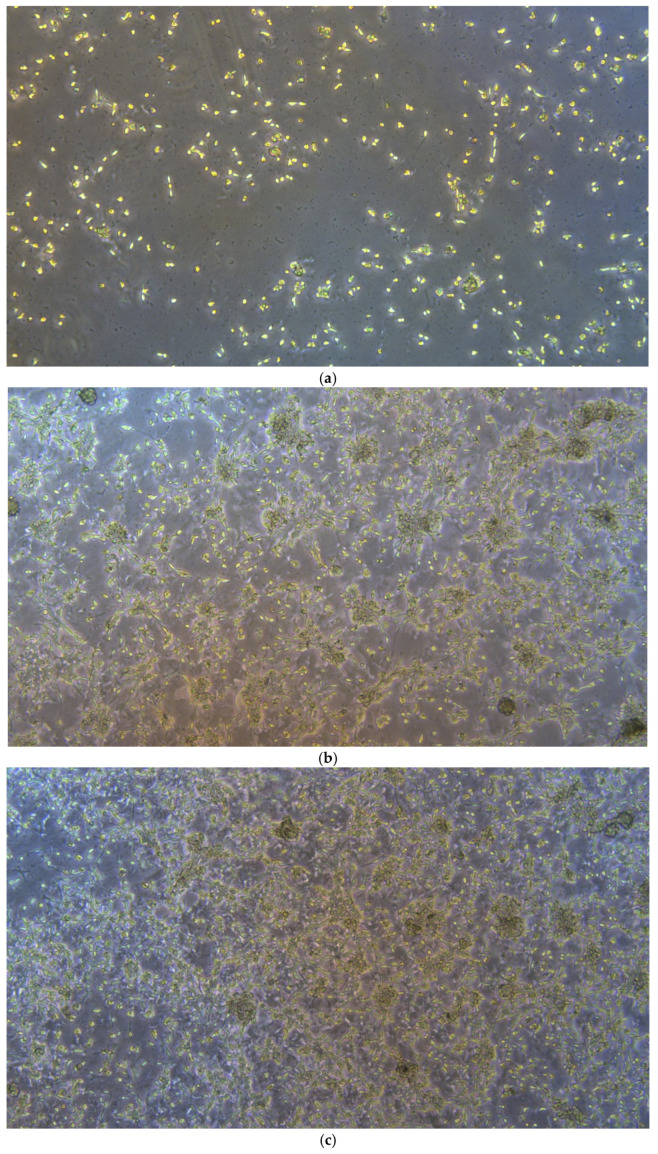

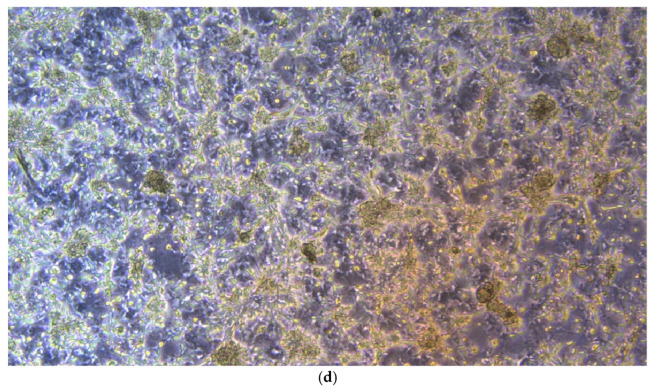
Micrographs of MRC-5 cell line growth on the surface of HSA-based hydrogels (50× magnification): (**a**) day 1; (**b**) day 2; (**c**) day 3; (**d**) day 4 of observation.

Analysis of growth dynamics demonstrated that MRC-5 fibroblasts effectively adhere to the surface of HSA-based hydrogels and retain their proliferative capacity (Figure 13). As early as 24 h post-seeding, individual attached cells exhibiting initial signs of morphological spreading were observed (Figure 13a). Over the subsequent 72 h, a progressive increase in cell density was recorded, confirming high cell viability and sustained proliferative activity in the presence of the hydrogel substrate (Figure 13b–d). These findings indicate that the developed hydrogels exhibit excellent biocompatibility, providing a microenvironment conducive to cell survival and proliferation. Notably, the growth pattern of MRC-5 cells on albumin-based hydrogels differed markedly from the classic monolayer typically formed on standard tissue culture plastic. Instead of spreading uniformly across the substrate, cells displayed a pronounced tendency to aggregate, forming compact three-dimensional structures resembling spheroid-like clusters. This morphological behavior suggests that the hydrogel exerts not merely a passive but an active regulatory influence on cellular organization, promoting a transition toward a 3D architecture. This phenomenon is likely driven by a combination of biochemical and biomechanical cues presented by the albumin matrix, which partially mimics key features of the native extracellular matrix (ECM). Consequently, the developed HSA-based hydrogels may be regarded not only as biocompatible but also as bioactive materials, capable of intentionally modulating cellular morphology and guiding tissue-like organization, properties that are highly desirable for advanced wound healing and regenerative medicine applications.

### 2.5. Investigation of the Rheological and Antibacterial Properties of Tetracycline-Loaded HSA-Based Hydrogels

**Figure 14:**
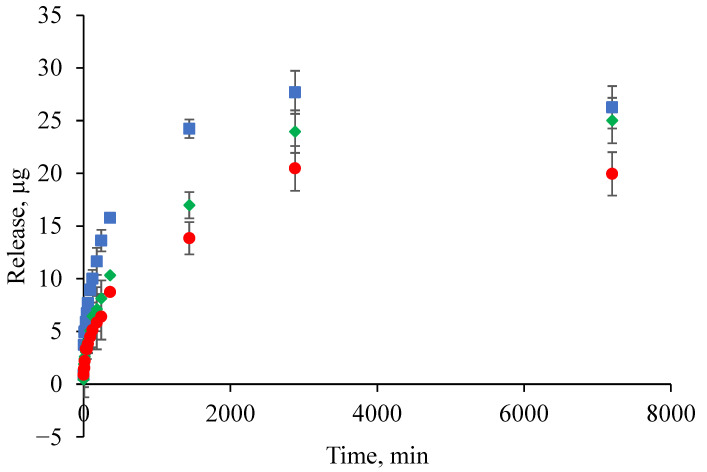
Release curves of tetracycline from human serum albumin-based hydrogels in PBS depending on HSA/EtOH ratio: 15 HSA–15 EtOH (blue squares), 20 HSA–15 EtOH (green rhombus), 15 HSA–10 EtOH (red circles).

To demonstrate the potential of HSA-based hydrogels for the controlled release of low molecular weight therapeutic drugs, a study was conducted on the kinetics of tetracycline release, a broad-spectrum antibiotic. The utilization of tetracycline as a model drug in this study is justified by a number of key reasons. Firstly, tetracycline exhibits a well-defined absorption peak in the UV region of the spectrum, thus enabling its concentration to be quantitatively determined in various media. This property, in turn, facilitates the study of the kinetics of its release from hydrogels. Secondly, tetracycline retains its biological activity when encapsulated in various materials. This ability makes it a convenient tool for evaluating the functional effectiveness of controlled release systems, including those based on hydrogels. Finally, the predictability of tetracycline’s behavior in laboratory conditions, that is to say, the absence of significant changes in its spectral properties or mechanisms of action, facilitates the comparison of different materials, environments, and release regimes. This versatility makes tetracycline a reliable model antibiotic for fundamental and applied research aimed at developing new biomedical materials, delivery systems, and antibacterial coatings. The antibiotic was loaded into the hydrogel at the gelation stage, and its final concentration was 1 mg/mL. The aforementioned assumption regarding the correlation between tetracycline release efficiency and the structural properties of the hydrogel is confirmed by a further experiment that examined the kinetics of tetracycline release from HSA-based hydrogels with varying HSA/EtOH ratios (Figure 14). As previously discussed, all HSA-based hydrogels exhibited a characteristic release pattern. Within the first hour, a rapid release was observed, the magnitude of which was dependent on the HSA/EtOH ratio: 15/15 HSA/EtOH ˃ 20/15 HSA/EtOH ˃ 15/10 HSA/EtOH. During the five-day monitoring period, the total amount of tetracycline released followed the same rank order. Consequently, an increase in the EtOH proportion during hydrogel preparation (i.e., a decrease in the HSA/EtOH ratio) resulted in accelerated and more substantial drug release, which is associated with the size of the pores formed in the hydrogel. According to the data obtained via scanning electron microscopy, an increase in the amount of denaturing agent, i.e., EtOH, results in an increase in the size of the pores in the hydrogel. This, in turn, affects the rate of drug release. The findings indicate that the HSA/EtOH ratio is a critical formulation parameter governing both the intensity of the initial release and the overall extent of tetracycline release from HSA-based hydrogels.

**Figure 15:**
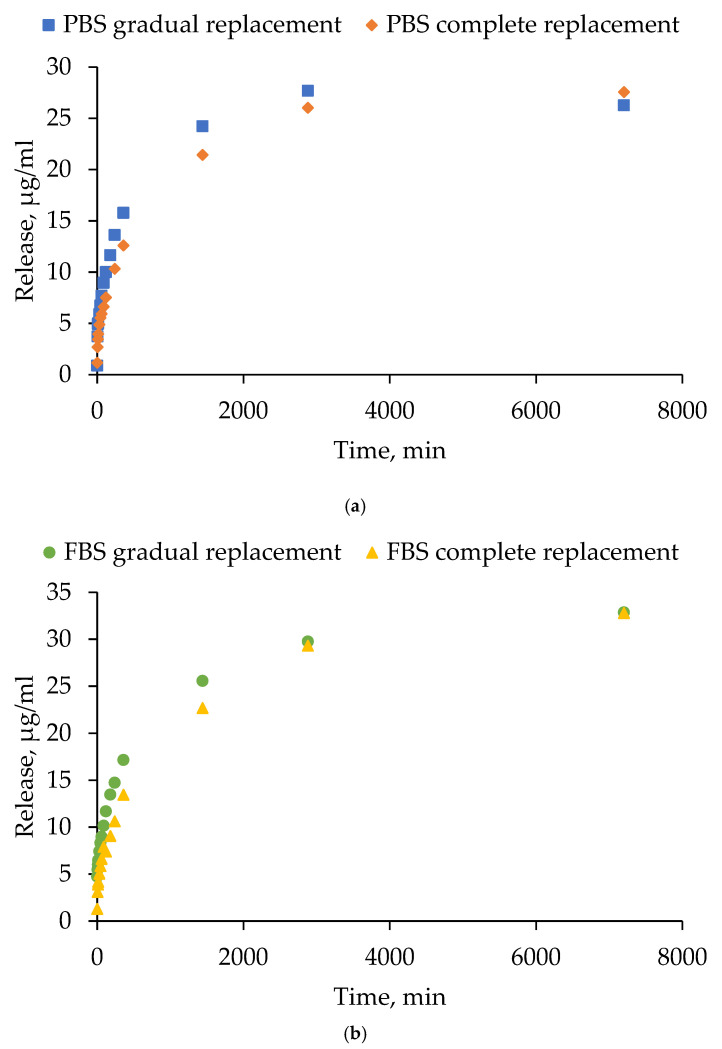
Release curves of tetracycline from human serum albumin-based hydrogels: (**a**) in PBS; (**b**) in FBS.

The efficiency of tetracycline release was studied in two media (phosphate-buffered saline (PBS) and 10% fetal bovine serum (FBS)) under different modes of medium replacement over the hydrogel (15 HSA–15 EtOH) surface (gradual and complete). The gradual replacement mode simulated partial fluid renewal (slow exchange, close to physiological), and the complete replacement mode simulated intensive washout. Figure 15 presents the antibiotic release curves in two different media: PBS (Figure 15a) and FBS (Figure 15b). The data points on the graphs represent the total amount of the substance (µg/mL) released from the hydrogel at the corresponding time point. Tetracycline release profile from HSA-based hydrogels exhibited two phases under all tested conditions, characterized by an initial rapid release during the first hour followed by a more gradual sustained release. Across both replacement modes, the final level of antibiotic release was found to be slightly higher in 10% FBS compared to PBS. This phenomenon is likely to be a consequence of the presence of proteins in FBS, which have the capacity to either bind to tetracycline, thereby improving its solubilization, or to enhance hydrogel disassembly. The mode of medium replacement exerted a negligible influence on the release kinetics in both PBS and 10% FBS. On the first day, the gradual replacement in the medium resulted in a slightly faster increase in tetracycline amount. However, these differences were subsequently leveled off at later time points. The findings suggest that tetracycline release is primarily governed by the internal diffusion and structural properties of the hydrogel, with medium composition exerting a stronger effect than the medium replacement mode. These findings emphasize the potential of human serum albumin-based hydrogel systems in the controlled release of tetracycline, with the capability to be adapted to a range of therapeutic requirements.

**Figure 16:**
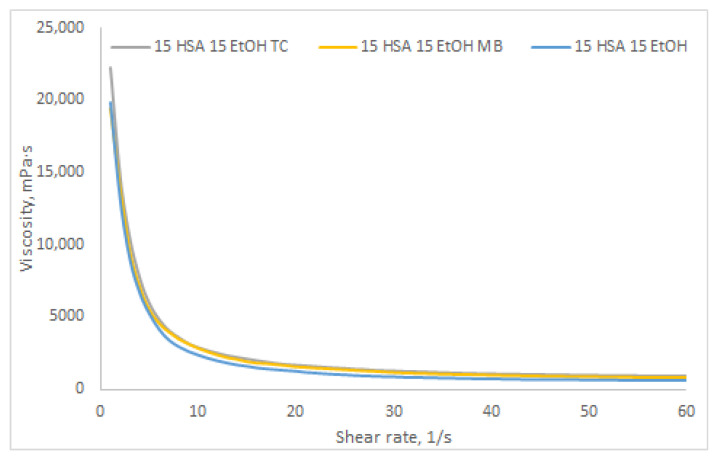
Dynamic viscosity curves for hydrogels composed of 15% *w*/*v* HSA–15% *v*/*v* EtOH (prepared at 60 °C for 10 min): blue line—blank HSA hydrogel; yellow line—HSA hydrogel loaded with methylene blue; gray line—HSA hydrogel loaded with tetracycline.

To investigate the influence of the nature of bioactive compounds on the rheological properties of HSA-based hydrogels, the dynamic viscosity of a 15% *w*/*v* HSA–15% *v*/*v* EtOH hydrogel with different antibacterial agents was measured (Figure 16). As antibacterial therapeutic agents, methylene blue and tetracycline were selected due to their significantly different chemical natures. Methylene blue is a highly hydrophilic compound with high water solubility [54], whereas tetracycline, despite its hydrophilic molecular structure, forms an intramolecular zwitterion salt that exhibits very low polarity and, consequently, poor solubility in polar solvents [55]. As shown in Figure 16, the dynamic viscosity curves reveal no statistically significant differences between HSA hydrogels loaded with either methylene blue or tetracycline and the “blank” HSA hydrogel. These findings suggest that the hydrogel formulation presented in this study—comprising two solvents, a strongly polar one (water) and an amphiphilic one (ethanol)—enables incorporation of both hydrophilic bioactive agents (dissolved in water) and more hydrophobic compounds (dissolved in ethanol), thus broadening its potential applications.

**Figure 17:**
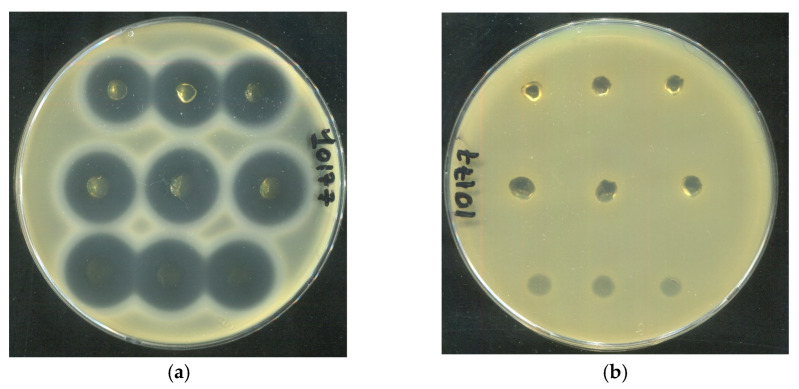

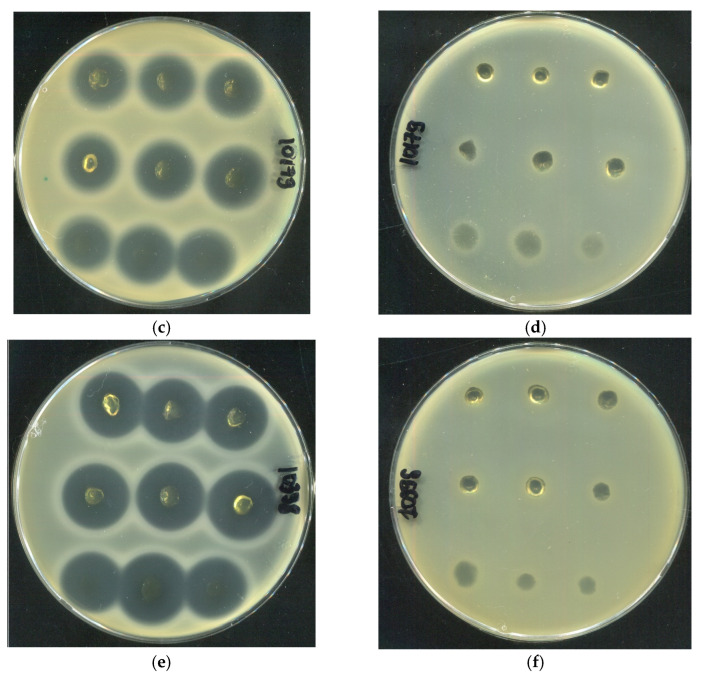
Assessment of the antibacterial activity of HSA-based hydrogels loaded with tetracycline (Top row on each Petri dish: hydrogel 20 HSA–15 EtOH (10 min); middle row: hydrogel 15 HSA–15 EtOH (10 min); bottom row: hydrogel 15 HSA–10 EtOH (30 min)). Panels (**a**,**c**,**e**): hydrogels containing tetracycline (1 mg/mL) applied onto agar plates inoculated with *S. aureus* strains SA10177 (**a**), SA10179 (**c**), and SA10398 (**e**); Panels (**b**,**d**,**f**): antibiotic-free hydrogels applied on the corresponding strains (negative controls). Inhibition zones are observed only in the presence of tetracycline, confirming the absence of intrinsic antibacterial activity of the HSA matrix.

The antibacterial activity of human serum albumin (HSA)-based hydrogels loaded with tetracycline (1 mg/mL) was evaluated against three clinical *Staphylococcus aureus* strains (SA10177, SA10179, and SA10398) isolated from wound surfaces of diverse etiologies. All tested formulations, including 20 HSA–15 EtOH (10 min), 15 HSA–15 EtOH (10 min), and 15 HSA–10 EtOH (30 min), produced distinct inhibition zones, confirming effective antibiotic delivery (Figure 17). In contrast, HSA hydrogels without tetracycline showed no inhibition of bacterial growth, ruling out any intrinsic antibacterial activity of the HSA matrix itself. Quantitative analysis of inhibition zone areas revealed a pronounced dependence of antibacterial efficacy on hydrogel composition (Table 10). According to the data in Table 10, no statistically significant differences were observed between the inhibition zone areas of the 15 HSA–15 EtOH (10 min) and 15 HSA–10 EtOH (30 min) formulations. In contrast, the 20 HSA–15 EtOH (10 min) hydrogel exhibited markedly lower antibacterial activity against all three tested strains. This reduced efficacy is consistent with scanning electron microscopy data indicating the formation of a denser, less porous network, which likely restricts tetracycline diffusion from the matrix (Figure 7c,d).

**Figure 18:**
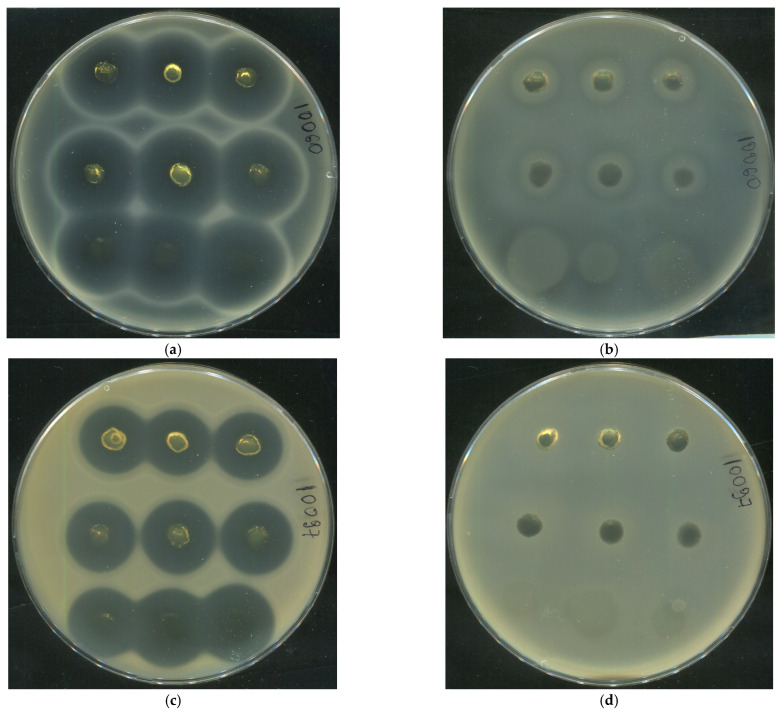

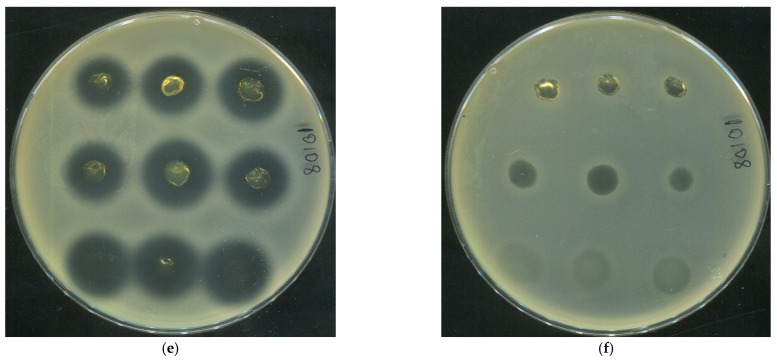
Assessment of the antibacterial activity of HSA-based hydrogels loaded with tetracycline (Top row on each Petri dish: hydrogel 20 HSA–15 EtOH (10 min); middle row: hydrogel 15 HSA–15 EtOH (10 min); bottom row: hydrogel 15 HSA–10 EtOH (30 min)). Panels (**a**,**c**,**e**): hydrogels containing tetracycline (1 mg/mL) applied onto agar plates inoculated with *S. epidermidis* strain SE10060 (**a**), *S. haemolyticus* strain SH10097 (**c**), *C. striatum* strain CS10108 (**e**); Panels (**b**,**d**,**f**): antibiotic-free hydrogels applied on the corresponding strains (negative controls). Inhibition zones are observed only in the presence of tetracycline, confirming the absence of intrinsic antibacterial activity of the HSA matrix.

To broaden the spectrum of Gram-positive pathogens clinically associated with wound infections, additional antibacterial assays were performed using human serum albumin (HSA)-based hydrogels loaded with tetracycline (1 mg/mL) against three clinically relevant strains: *Staphylococcus epidermidis* (SE10060), *Staphylococcus haemolyticus* (SH10097), and *Corynebacterium striatum* (CS10108). In all cases, well-defined inhibition zones were observed, whereas antibiotic-free hydrogels exhibited no inhibitory effect (Figure 18), confirming the absence of intrinsic antibacterial activity of the HSA matrix. The largest inhibition zone was recorded for *S. epidermidis*, while *C. striatum* showed the lowest susceptibility across all tested formulations (Table 11). Notably, the densest hydrogel (20 HSA–15 EtOH) demonstrated the weakest antibacterial activity against all strains, whereas the 15 HSA–15 EtOH and 15 HSA–10 EtOH formulations showed no statistically significant differences in bacterial growth inhibition. This finding aligns with tetracycline release kinetics in PBS (Figure 14), suggesting more efficient antibiotic diffusion from less densely cross-linked matrices. Thus, both formulations—15 HSA–15 EtOH and 15 HSA–10 EtOH—can be regarded as promising platforms for topical wound therapy: the former as a viscous, non-injectable dressing, and the latter as an injectable formulation. These results confirm that the developed HSA-based hydrogels effectively release tetracycline at biologically active concentrations, providing robust inhibition of key Gram-positive wound pathogens, including staphylococci and corynebacteria, and thereby demonstrating strong potential for use in polymicrobial wound infections.

## 3. Conclusions

In this study, a method for preparing human serum albumin-based hydrogels via thermally induced gelation was developed. It was found that aqueous HSA hydrogels form at temperatures significantly above the denaturation temperature of human serum albumin, while the addition of ethanol significantly reduces the gelation temperature. It was demonstrated that the combination of phosphate-buffered saline (PBS) as an albumin solvent and ethyl alcohol enables the formation of hydrogels exhibiting optical transparency and mechanical stability. In the PBS-HSA-EtOH system, the ideal compositions for hydrogel formation are 10% HSA–20% EtOH and 15% HSA–20% EtOH at 55 °C with a 10 min incubation time; 10% HSA–15% EtOH and 15% HSA–15% EtOH at 60 °C with a 10 min incubation time. Dynamic light scattering (DLS) revealed that hydrogel formation in aqueous HSA solutions results in significantly larger albumin aggregates (Z-average = 1500–8500 nm) compared to hydrogels based on HSA solutions in PBS (Z-average = 450–500 nm). It was demonstrated that hydrogels prepared via thermally induced gelation with a denaturing agent (ethyl alcohol) exhibit dynamic viscosity in the range of 100–10,000 mPa·s. The developed HSA-based hydrogels exhibit biodegradability in biologically relevant media, demonstrating gradual degradation over five days of incubation. Their biocompatibility was confirmed by MTT assay results, which showed no significant reduction in fibroblast viability even at high hydrogel concentrations. Moreover, the hydrogels actively supported fibroblast adhesion and sustained subsequent proliferative activity. The release kinetics of tetracycline from human serum albumin-based hydrogels were investigated in two media: PBS and FBS. The hydrogels exhibited differential behavior in the studied media: they maintained structural integrity in PBS, while softening and color changes were observed in FBS, indicating biodegradation under near-physiological conditions. All tested formulations of human serum albumin-based hydrogels loaded with tetracycline (1 mg/mL) was demonstrated antibacterial activity of against *Staphylococcus aureus*, *Staphylococcus epidermidis*, *Staphylococcus haemolyticus*, and *Corynebacterium striatum* strains isolated from wound surfaces of diverse etiologies. The resultant human serum albumin-based hydrogels represent promising platforms for developing topical formulations for wound healing and tissue engineering applications.

## 4. Materials and Methods

### 4.1. Materials

Lyophilized human serum albumin (REANHAL, Budapest, Hungary) was used without further purification. Ethanol (EtOH) (Kemerovo Pharmaceutical Factory, Kemerovo, Russian Federation) underwent rectification-based dehydration under laboratory conditions to obtain the absolute product. Type 1 water was purified using a Milli-Q water purification system (Merck Millipore, Billyrick, MA, USA). Tablet-form Dulbecco’s phosphate-buffered saline (PBS) was supplied by Sigma-Aldrich, St. Louis, MI, USA, fetal bovine serum (FBS) (Thermo Fisher Scientific, Waltham, MA, USA), tetracycline hydrochloride (Gerbu biotechnik, Heidelberg, Germany). HSA modified by sulfo-Cy5 dye (HSA-Cy5) was obtained according to [56].

### 4.2. Synthesis of Hydrogels Based on Human Serum Albumin

Synthesis of HSA hydrogels was carried out by thermally induced gelation. For the formation of HSA hydrogels, human serum albumin was dissolved in deionized water or Dulbecco’s phosphate-buffered saline to obtain solutions with a mass fraction of 10–20%. After complete dissolution of HSA, a required volume of ethanol was added to the solution. The gel was subsequently formed from the resulting solution via static heating. The formation of HSA-based hydrogels was visually assessed using the vial inversion method. All hydrogels were synthesized under strictly sterile conditions.

### 4.3. In Situ Study of Gelation in HSA-Based Aqueous-Ethanol Systems

Measurements of gelation onset time and mean aggregate size during gelation were performed using dynamic light scattering (DLS) on a Zetasizer Nano ZC particle size and zeta potential analyzer (Malvern Instruments, Malvern, UK). Gelation measurements were conducted in a ZEN0040 cuvette with a lid (Malvern Instruments, Malvern, UK), with an added liquid volume of 500 μL. For each sample, 150 measurements were performed in “size” mode, with a 0 s delay between measurements, 5 runs per measurement, and a run duration of 5 s. Gelation measurements were initiated only after reaching the gelation temperature (60 °C).

### 4.4. Determination of the Secondary Structures Content in HSA Molecule by Circular Dichroism

Circular dichroism spectra were recorded using a J-600 spectropolarimeter (Jasco, Hachioji, Japan) at room temperature in the wavelength range corresponding to the far UV region (λ = 190–240 nm) in a quartz cuvette with an optical path length of 0.01 cm. The volume of the studied sample was 120 μL, the protein concentration in the sample was 1.5 × 10^−5^ M. For each spectrum, ten spectra were accumulated at a scanning speed of 50 nm/min. The obtained spectra were processed by extrapolation from theoretical spectra, and the percentage of secondary structures in the protein molecule was found from the obtained data.

### 4.5. Lyophilization of HSA-Based Hydrogels

Lyophilization of hydrogels was performed using a BK-FD10S freeze dryer (Biobase, Jinan, China). The temperature of the cooling condenser in the dryer was set at −60 °C. The samples were cooled in the condenser to −25 °C and subsequently subjected to vacuum drying. After initiating the vacuum, the samples were heated to 0 °C over a period of 20 min and then dried to room temperature for 10 h. The product temperature was calculated based on the reading from a temperature probe.

### 4.6. Study of Lyophilizated HSA-Based Hydrogels Microstructure

The microstructure of the samples surface was studied by scanning electron microscopy (SEM). The samples were fixed on a sample holder using double-sided carbon tape, coated with 10 nm gold/palladium layer and analyzed using an EVO 10 scanning electron microscope (Carl Zeiss AG, Oberkochen, Germany) at an accelerating voltage of 10 kV.

### 4.7. Measurement of Dynamic Viscosity of HSA-Based Hydrogels

In this study, the dynamic viscosity was measured using a DV3T-RV viscometer (Brookfield Engineering Labs, Middleboro, MA, USA) with a cone/plate geometry at 25 °C, utilizing the RheocalcT software v1.2.19 package. Depending on the paste viscosity, CPE-40 and CPE-52 spindles were employed during measurements.

### 4.8. Study of the Stability of the Human Serum Albumin-Based Hydrogels in Biologically Relevant Media

The study of the stability of the human serum albumin-based hydrogels was conducted at 37 °C, with constant stirring at a speed of 300 rpm. For the experiment, a hydrogel containing 15% albumin by weight and 15% ethanol by volume was prepared, to which 1 mL of PBS or 10% FBS was added. For experiments in FBS, Cy5-labeled albumin (10% of the total amount of albumin added) was added during the preparation of the hydrogel. Two approaches were used to evaluate the effect of the medium replacement mode:

- Gradual medium replacement: after adding 1 mL of PBS/FBS to the hydrogel, 100 µL of supernatant was removed from the solution at specified time points and replaced with an equal volume of fresh PBS/FBS.
- Complete medium replacement: the procedure was similar, but 900 µL of supernatant was collected and replaced with an equal volume of fresh PBS/FBS. Samples were collected at the following time points: 0 min, 5 min, 10 min, 15 min, 30 min, 45 min, 60 min, 120 min, 180 min, 240 min, 360 min, 24 h, 48 h and 120 h. The samples were analyzed for tetracycline content using a spectrophotometric method at room temperature, with an absorption wavelength of λ_abs_ = 280 nm or λ_abs_ = 650 nm (for Cy5-HSA). The result was recorded as the absorption value at the end point.

### 4.9. Cytotoxicity Study of HSA-Based Hydrogels

The cytotoxicity of HSA-based hydrogels was evaluated using the MTT (3-(4,5-dimethylthiazol-2-yl)-2,5-diphenyltetrazolium bromide) colorimetric assay. Experiments were carried out on the MRC-5 diploid human lung fibroblast cell line. Cells were seeded in 96-well plates at a density of 4 × 10^3^ cells per well and cultured in Dulbecco’s Modified Eagle’s Medium (DMEM) supplemented with 10% fetal bovine serum (Thermo Fisher Scientific, Waltham, MA, USA), 100 U/mL penicillin, and 100 µg/mL streptomycin, under standard conditions (37 °C, 5% CO_2_, humidified atmosphere). Following an overnight attachment period, the culture medium was replaced with fresh medium containing the HSA hydrogel, and cells were incubated for 48 h under the same conditions. Untreated cells cultured in complete medium served as the negative control. After incubation, the medium was aspirated, and 10 µL of MTT solution (25 mg/mL in PBS) was added to each well. The plates were incubated for 4 h at 37 °C to allow formazan crystal formation. Subsequently, the MTT solution was removed, and the resulting formazan crystals were solubilized in 100 µL of dimethyl sulfoxide (DMSO). Absorbance was measured at 570 nm (with a reference wavelength of 620 nm) using a Clariostar microplate reader (BMG Labtech, Ortenberg, Germany). All experiments were performed in at least triplicate, and data are presented as mean ± standard deviation.

### 4.10. Biocompatibility Assessment of Human Serum Albumin-Based Hydrogels

HSA-based hydrogels intended for biocompatibility evaluation were fabricated by dispensing 500 µL of a solution containing HSA, PBS and EtOH into sterile 30 mm Petri dishes. The dishes were sealed with adhesive PCR plate sealing film and subjected to thermal crosslinking in a thermocycler (Eppendorf, Hamburg, Germany) at 60 °C for 20 min to promote hydrogel formation. Following gelation, the hydrogels were stored at 4 °C until further use. Prior to cell-based assays, all hydrogels underwent surface sterilization via UV irradiation for 20 min. MRC-5 diploid human lung fibroblast cell line was used to determine the possibility of cell proliferation on the surface of the hydrogel. Hydrogel-coated Petri dish was incubated in 2 mL DMEM supplemented with 10% fetal bovine serum (FBS), 1% GlutaMax and 1% antibiotic-antimycotic for 24 h in CO_2_-incubator. After that the medium was removed, and 3 × 10^5^ cells were seeded and cultured for 4 days (at 37 °C and 5% CO_2_).

### 4.11. Synthesis of Human Serum Albumin-Based Hydrogels with Tetracycline

The synthesis of hydrogels based on human serum albumin (HSA) containing tetracycline was carried out using the thermally induced gelation method. The HSA protein was dissolved in PBS to obtain a solution with a mass fraction of 15%. Following the complete dissolution of the albumin, the requisite volume of tetracycline solution in ethanol (10 mg/mL) was introduced into the system to achieve an ethanol volume fraction of 15%. The mixture was stirred until a homogeneous solution was obtained, ensuring uniform distribution of tetracycline throughout the volume. The resulting solution was then subjected to static heating at 60 °C for 10 min in order to form an HSA–tetracycline hydrogel. The assessment of gel formation and integrity was conducted through the utilization of the vial inversion method. The incorporation of tetracycline into the hydrogel structure was observed by the change in color of the sample.

### 4.12. Study of the Kinetics of Tetracycline Release from Hydrogels Based on Human Serum Albumin

The release of tetracycline from the HSA-hydrogel was conducted at 37 °C, with constant stirring at a speed of 300 rpm. For the experiment, a hydrogel containing a known amount of tetracycline was utilized, to which 1 mL of PBS (pH 7.4) or 10% *w*/*v* FBS was added. Two approaches were used to evaluate the effect of the medium replacement mode:

- Gradual medium replacement: similar to 4.8.
- Complete medium replacement: the procedure was similar, but 900 µL of supernatant was collected and replaced with an equal volume of fresh PBS/FBS. Samples were collected at the following time points: 0 min, 5 min, 10 min, 15 min, 30 min, 45 min, 60 min, 120 min, 180 min, 240 min, 360 min, 24 h, 48 h and 120 h.

The samples were analyzed for tetracycline content using a spectrophotometric method at room temperature, with an absorption wavelength of λ_abs_ = 370 nm. The result was recorded as the absorption value at the end point.

### 4.13. Antibacterial Activity of Tetracycline-Loaded Hydrogels Based on Human Serum Albumin

Bacterial strains used for evaluating antibacterial activity were kindly provided by the Center for Collective Use “Collection of Extremophilic Microorganisms and Type Cultures” Institute of Chemical Biology and Fundamental Medicine, Siberian Branch of the Russian Academy of Sciences (Novosibirsk, Russia). Six tetracycline-susceptible strains—*Staphylococcus aureus* (SA10177, SA10179, and SA10398), *Staphylococcus epidermidis* (SE10060), *Staphylococcus haemolyticus* (SH10097), and *Corynebacterium striatum* (CS10108)—were employed in this study. All strains are nosocomial isolates derived from wounds of diverse etiologies. The antibacterial activity of human serum albumin based hydrogels was assessed using the agar diffusion method. Sterile Petri dishes were prepared with a bottom layer of nutrient agar (microbial culture broth, Microgen, Nizhny Novgorod, Russian Federation), followed by a top layer of LB agar (Servicebio, Wuhan, China) inoculated with a standardized suspension of *S. aureus* (~10^6^ CFU/mL). Approximately 70 µL of hydrogel loaded with tetracycline (1 mg/mL) was carefully applied onto the surface of the agar. Hydrogels without tetracycline served as negative controls. All plates were incubated at 37 °C for 18–24 h in a controlled-temperature incubator. The antibacterial effect was evaluated by measuring the diameter and area of the growth inhibition zone surrounding each hydrogel deposit. The inhibition zone area was quantified using ImageJ software (version 1.53t, National Institutes of Health, Bethesda, MD, USA) based on standardized digital images captured under uniform lighting conditions.

## Figures and Tables

**Table 1:** Schematic representation of hydrogel formation as a function of temperature and albumin concentration in aqueous solution. Red denotes conditions yielding no gelation; orange indicates incomplete gelation; yellow signifies hydrogel formation with substantial HSA denaturation.

HSA (*w*/*v*)/T (°C)	60	65	70	75	80
10					
15					
20					

**Table 2:** Calculation of the number of α-helices and β-sheets in human serum albumin solutions using circular dichroism data.

Sample	α-Helices	β-Sheets
HSA (10 mM PBS, pH 7.4)	61.1 ± 0.9	3.2 ± 0.1
HSA (50% EtOH)	54.3 ± 0.8	5.6 ± 0.2

**Table 3:** Schematic representation of hydrogel formation data as a function of temperature, HSA mass fraction, and ethanol volume fraction. Red cells indicate unformed hydrogel; orange cells correspond to partially formed hydrogel; green cells correspond to formed hydrogel; yellow cells represent hydrogel formed with substantial denaturation of HSA.

	55 °C			60 °C		
HSA (*w*/*v*)/EtOH (*v*/*v*)	10	15	20	10	15	20
0						
5						
10						
15						
20						

**Table 4:** Summary data on hydrogel formation depending on incubation time, HSA mass fraction, and ethanol volume fraction at 60 °C (for 0, 5, and 10% *v*/*v* ethanol with incubation times of 10, 30, and 60 min). The red frame corresponds to unformed hydrogel; green frames indicate formed hydrogel.

	10 *w*/*v* HSA			15 *w*/*v* HSA			20 *w*/*v* HSA		
EtOH (*v*/*v*)/t (min)	10	15	20	10	15	20	10	15	20
10									
30									
60									

**Table 5:** Summary data on hydrogel formation depending on incubation time, HSA mass fraction, and ethanol volume fraction at 60 °C (for 10, 15, and 20% *v*/*v* ethanol with incubation times of 1, 5, and 10 min). The red frame corresponds to unformed hydrogel; the yellow frame represents hydrogel formed with substantial denaturation of HSA; green frames indicate formed hydrogel.

	10 *w*/*v* HSA			15 *w*/*v* HSA			20 *w*/*v* HSA		
EtOH (*v*/*v*)/t (min)	10	15	20	10	15	20	10	15	20
1									
5									
10									

**Table 6:** Summary of hydrogel formation as a function of ethanol volume fraction based on DLS measurements (HSA concentration: 10% *w*/*v*; temperature: 60 °C).

	PBS			Water		
Ethanol (*v*/*v*, %)	Hydrogel Formation (Yes/No)	Gelation Onset Time (s)	Average Hydrodynamic Diameter After Gelation (nm)	Hydrogel Formation (Yes/No))	Gelation Onset Time (s)	Average Hydrodynamic Diameter After Gelation (nm)
0	No	-	-	No	-	-
5	No	-	-	Yes	420	1435 ± 826
10	No	-	-	Yes	336	5845 ± 3945
15	Yes	2700	443 ± 170	Yes	210	8524 ± 5284
20	Yes	600	405 ± 159	Yes	156	8424 ± 7585

**Table 7:** Summary of dynamic viscosity values for HSA-based hydrogels at shear rates of 5, 15, and 45 s^−1^.

Hydrogel System	Dynamic Viscosity at a Shear Rate of 5 s^−1^, mPa·s	Dynamic Viscosity at a Shear Rate of 15 s^−1^, mPa·s	Dynamic Viscosity at a Shear Rate of 45 s^−1^, mPa·s
20 HSA–20 EtOH—60 °C	1 min—unmeasurable	1 min—unmeasurable	1 min—unmeasurable
	5 min—unmeasurable	5 min—unmeasurable	5 min—unmeasurable
	10 min—unmeasurable	10 min—unmeasurable	10 min—unmeasurable
20 HSA–15 EtOH—60 °C	1 min—2818	1 min—1164	1 min—732
	5 min—7660	5 min—2500	5 min—1129
	10 min—6787	10 min—2474	10 min—1191
20 HSA–10 EtOH—60 °C	10 min—25.57	10 min—15.7	10 min—12.53
	30 min—2223	30 min—1072	30 min—621.8
	60 min—3889	60 min—1495	60 min—815.8
15 HSA–20 EtOH—60 °C	1 min—7342	1 min—2183	1 min—930.5
	5 min—23,290	5 min—10,850	5 min—4379
	10 min—unmeasurable	10 min—unmeasurable	10 min—unmeasurable
15 HSA–15 EtOH—60 °C	1 min—1071	1 min—398.9	1 min—288.3
	5 min—3373	5 min—1244	5 min—590.9
	10 min—5239	10 min—1627	10 min—714.4
15 HSA–10 EtOH—60 °C	10 min—10.25	10 min—7.85	10 min—6.54
	30 min—1177	30 min—413.7	30 min—257.8
	60 min—2542	60 min—487.2	60 min—252.3
10 HSA–20 EtOH—60 °C	1 min—3651	1 min—1177	1 min—515.9
	5 min—19,760	5 min—4868	5 min—2011
	10 min—32,070	10 min—7647	10 min—3197
10 HSA–15 EtOH—60 °C	1 min—344	1 min—153.1	1 min—8.18
	5 min—754.1	5 min—310.6	5 min—8.17
	10 min—2024	10 min—1619	10 min—24.52
10 HSA–10 EtOH—60 °C	10 min—6.54	10 min—3.27	10 min—3.27
	30 min—139	30 min—57.22	30 min—31.07
	60 min—605	60 min—237.1	60 min—139.5

**Table 8:** Ranges of dynamic viscosity for human serum albumin-based hydrogels.

Group	Samples Included in the Group	Dynamic Viscosity Range at 3 s^−1^ (mPa·s)	Dynamic Viscosity Range at 15 s^−1^ (mPa·s)	Dynamic Viscosity Range at 45 s^−1^ (mPa·s)
1	10 HSA–10 EtOH (30 min, 60 min) 10 HSA–15 EtOH (10 min, 30 min)	100–1000	50–300	20–150
2	10 HSA–15 EtOH (10 min) 10 HSA–20 EtOH (1 min) 15 HSA–10 EtOH (30 min, 60 min) 15 HSA–15 EtOH (1 min, 5 min) 20 HSA–10 EtOH (30 min, 60 min) 20 HSA–15 EtOH (1 min)	1000–5000	300–1500	150–300
3	15 HSA–15 EtOH (10 min) 15 HSA–20 EtOH (1 min) 20 HSA–15 EtOH (5 min, 10 min)	5000–10,000	1500–2500	300–1200
4	10 HSA–20 EtOH (5 min) 15 HSA–20 EtOH (5 min, 10 min)	10,000–25,000	2500–10,000	1200–4500

**Table 9:** Dynamic viscosity ratios (Thixotropic Index) at different shear rates for HSA-based hydrogels.

Hydrogel System	The Ratio of the Dynamic Viscosities at Shear Rates of 5 s^−1^ and 15 s^−1^	The Ratio of the Dynamic Viscosities at Shear Rates of 5 s^−1^ and 45 s^−1^
20 HSA–20 EtOH—60 °C	1 min—unmeasurable	1 min—unmeasurable
	5 min—unmeasurable	5 min—unmeasurable
	10 min—unmeasurable	10 min—unmeasurable
20 HSA–15 EtOH—60 °C	1 min—2.42	1 min—3.85
	5 min—3.06	5 min—6.78
	10 min—2.74	10 min—5.70
20 HSA–10 EtOH—60 °C	10 min—1.63	10 min—2.04
	30 min—2.07	30 min—3.58
	60 min—2.60	60 min—4.76
15 HSA–20 EtOH—60 °C	1 min—3.36	1 min—7.89
	5 min—2.15	5 min—5.32
	10 min—unmeasurable	10 min—unmeasurable
15 HSA–15 EtOH—60 °C	1 min—2.68	1 min—3.71
	5 min—2.71	5 min—5.71
	10 min—3.22	10 min—7.33
15 HSA–10 EtOH—60 °C	10 min—1.31	10 min—1.56
	30 min—2.85	30 min—4.56
	60 min—5.22	60 min—10.1
10 HSA–20 EtOH—60 °C	1 min—3.10	1 min—7.08
	5 min—4.06	5 min—9.82
	10 min—4.19	10 min—10.03
10 HSA–15 EtOH—60 °C	1 min—2.25	1 min—42.5
	5 min—2.43	5 min—92.3
	10 min—1.25	10 min—82.5
10 HSA–10 EtOH—60 °C	10 min—2.00	10 min—2.00
	30 min—2.43	30 min—4.47
	60 min—2.55	60 min—4.33

**Table 10:** Inhibition zone areas (cm^2^) against *S. aureus* strains induced by HSA-based hydrogels loaded with tetracycline (1 mg/mL).

	SA10177	SA10179	SA10398
	Inhibition Zone Area (cm^2^)		
20 HSA–15 EtOH—10 min	3.38 ± 0.10	2.57 ± 0.05	2.87 ± 0.06
15 HSA–15 EtOH—10 min	4.36 ± 0.16	2.93 ± 0.18	3.15 ± 0.08
15 HSA–10 EtOH—30 min	3.90 ± 0.06	2.66 ± 0.10	2.83 ± 0.39

**Table 11:** Inhibition zone areas (cm^2^) against *S. epidermidis*, *S. haemolyticus*, *C. striatum* strains induced by HSA-based hydrogels loaded with tetracycline (1 mg/mL).

	SE10060	SH10097	CS10108
	Inhibition Zone Area (cm^2^)		
20 HSA–15 EtOH—10 min	3.64 ± 0.16	2.51 ± 0.05	1.88 ± 0.25
15 HSA–15 EtOH—10 min	4.11 ± 0.06	2.93 ± 0.19	2.15 ± 0.28
15 HSA–10 EtOH—30 min	3.90 ± 0.37	3.11 ± 0.29	2.12 ± 0.08

## Data Availability

The data presented in this study are openly available in article.

## Abbreviations

The following abbreviations are used in this manuscript:

HSA	human serum albumin
BSA	bovine serum albumin
DLS	dynamic light scattering
FBS	fetal bovine serum
PBS	phosphate-buffered saline
